# Phytohormones involved in vascular cambium activity in woods: current progress and future challenges

**DOI:** 10.3389/fpls.2024.1508242

**Published:** 2024-12-17

**Authors:** Wenjing Ding, Chencan Wang, Man Mei, Xiaoxu Li, Yuqian Zhang, Hongxia Lin, Yang Li, Zhiqiang Ma, Jianwei Han, Xiaoxia Song, Minjie Wu, Caixia Zheng, Jinxing Lin, Yuanyuan Zhao

**Affiliations:** ^1^ College of Biological Sciences and Biotechnology, Beijing Forestry University, Beijing, China; ^2^ Beijing Advanced Innovation Center for Tree Breeding by Molecular Design, Beijing Forestry University, Beijing, China; ^3^ National Engineering Research Center for Forest Breeding and Ecological Restoration, Beijing Forestry University, Beijing, China; ^4^ State Key Laboratory of Tree Genetics and Breeding, College of Biological Sciences and Technology, Beijing Forestry University, Beijing, China; ^5^ State Key Laboratory of Efficient Production of Forest Resources, Beijing Forestry University, Beijing, China; ^6^ Plant Protection Institute, Hebei Academy of Agriculture and Forestry Sciences, Baoding, Hebei, China; ^7^ China National Tree Seed Group Corporation Limited, Beijing, China; ^8^ China Forestry (Sanming) Development Corporation Limited, Sanming, Fujian, China

**Keywords:** wood, vascular cambium, phytohormones, mutual effect, growth and development

## Abstract

Vascular cambium is the continuation of meristem activity at the top of plants, which promotes lateral growth of plants. The vascular cambium evolved as an adaptation for secondary growth, initially in early seed plants, and became more refined in the evolution of gymnosperms and angiosperms. In angiosperms, it is crucial for plant growth and wood formation. The vascular cambium is regulated by a complex interplay of phytohormones, which are chemical messengers that coordinate various aspects of plant growth and development. This paper synthesizes the current knowledge on the regulatory effects of primary plant hormones and peptide signals on the development of the cambium in forest trees, and it outlines the current research status and future directions in this field. Understanding these regulatory mechanisms holds significant potential for enhancing our ability to manage and cultivate forest tree species in changing environmental conditions.

## Introduction

Plants, especially woody plants, accumulate biomass year after year through longitudinal elongation growth and radial thickening growth, which is plays an important role in production and life (Wang et al., 2021). Wood is the result of continuous deposition and accumulation of secondary xylem produced by the proliferation and differentiation of cells in the vascular cambium, and vascular stem cells proliferate and differentiate to produce secondary xylem and secondary phloem, which transport water and photosynthetic products respectively to support the vertical growth of plants (Sun et al., 2023). The regulation of proliferation and differentiation of vascular cambium has always been a key problem in plant biology and forestry research. In recent years, through the long-term study of model plants *Arabidopsis thaliana* and *Populus*, the molecular mechanism of vascular cambium development has made great progress, and most of the research results have been verified in various species, indicating that there is a conservative regulation mechanism of vascular cambium activity among species (Etchells et al., 2015). The proliferation and differentiation of vascular cambium are regulated by a variety of signals, including long-distance hormone signals from apical meristem and polypeptide signals from surrounding tissues, a large number of transcription factors and MicroRNAs (miRNAs). Their signal pathways interact and coordinate with each other to jointly regulate the activity of vascular cambium.

The vascular cambium is regulated by a complex interplay of phytohormones, which are chemical messengers that coordinate various aspects of plant growth and development (Amara et al., 2022; Kumar et al., 2023). Auxin in the vascular cambium region promotes cell division and differentiation, thereby maintaining the homeostasis and expansion of vascular tissues. Polar transport of auxin is dependent on the regulation of the PIN family of proteins, and the expression and localization of these proteins in the vascular cambium is critical for the formation of auxin gradient (Yang et al., 2021). Abscisic acid (ABA), on the other hand, acts mainly under abiotic stress conditions. is able to inhibit the activity of the vascular cambium and reduce ABA synthesis by regulating miRNAs and MYB transcription factors (Nguyen et al., 2023).Thus there is a complex interaction between ABA and growth hormones that jointly regulate the activity and differentiation of the vascular cambium.

At the same time, these stresses disrupt the metabolic balance of reactive oxygen species (ROS) in plants (Ravi et al., 2023), ROS can cause oxidative damage to cellular components, including lipids, proteins, and DNA, leading to disruption of cellular functions and ultimately inhibiting the division and differentiation of cambial cells. In this context, phytohormones play a critical role in mitigating the negative effects of ROS on the vascular cambium (Zinta et al., 2016; Lohani et al., 2020). For instance, auxins can upregulate the expression of antioxidant enzymes, such as superoxide dismutase and catalase, which scavenge ROS and protect the cambial cells from oxidative damage (Das et al., 2024; Mittal et al., 2024), Additionally, ethylene signaling promotes cell division in the vascular formation layer by inducing the expression of ethylene response factors (ERFs) (Hellmann et al., 2018). Gibberellin (GA) also plays a key role in vascular formation, and its level is higher in the vascular-forming region, which is associated with the expansion of vascular cambium cells (Miyashima et al., 2013). Therefore, phytohormones play an important role in maintaining ROS homeostasis and regulating vascular cambium activity, which is essential for plants to cope with environmental stress and maintain normal growth and development.

The vascular cambium is the power center for stem thickening and wood accumulation (Du et al., 2023), and its activity directly affects wood yield and quality (Wang, 2024). Besides, the study of vascular cambium also provides a scientific basis for the development of forest carbon sinks and forest management strategies (Gričar, 2012), and in-depth study of vascular cambium also promotes the development of biotechnology. Given this context, our study focuses on the research progress of plant hormones in the regulation of vascular cambium activity in recent years, and the potential application of hormone signaling pathway in the development of vascular cambium are emphatically introduced. Therefore, it is hoped that it will be complementary to the network that regulates the characterization and activity of the vascular formation layer in forest trees.

## Significance of vascular cambium in plant growth and development

In higher plants, plants need to transfer sugar synthesized in the aboveground stems and leaves to roots, and transport water and minerals absorbed by roots to aboveground tissues. In order to accomplish this function, plants have formed a complex vascular system in the process of evolution, and its appearance was a necessary condition for land plants to land successfully. The formation process of vascular tissue is an orderly plant development process, which can flexibly adjust the environment change. The development of vascular tissue in plants can be divided into three stages: the beginning of vascular tissue, the maintenance of vascular tissue stem cells and the differentiation of vascular tissue. All vascular tissues of plants come from four primitive cells in the stage of spherical embryo. They produce protovascular tissue cells through asymmetric division, and further differentiate into various vascular cells, including xylem cells, phloem cells and procambium cells. Procambium cells can produce xylem cells, phloem cells and cambium cells, while cambium cells can continue to secondary growth, so procambium cells and cambium cells are considered to have the function of vascular cambium stem cells.

**Figure 1:**
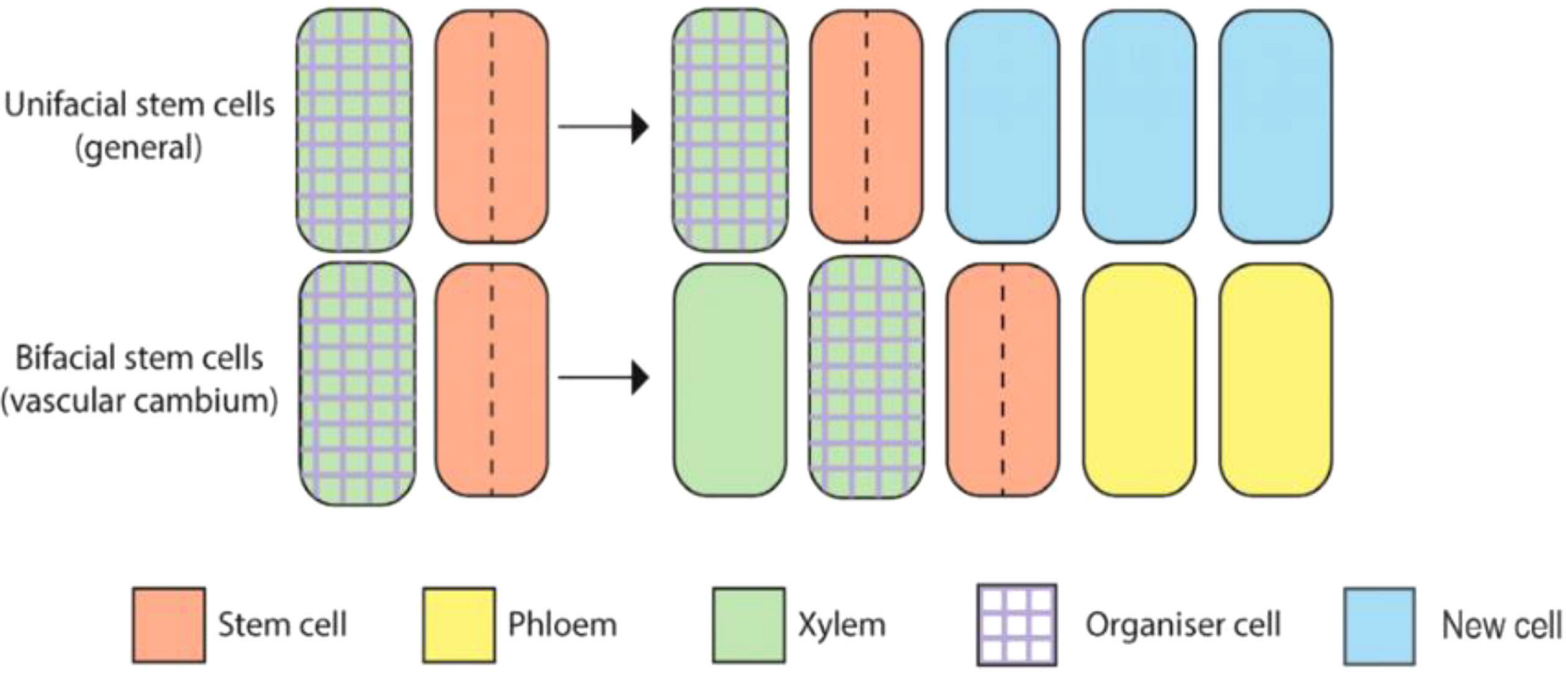
Graphical overview of vascular cambium dynamics in *Arabidopsis*.

Vascular cambium are bifacial stem cells, also known as pluripotent stem cells (Sun et al., 2023), with the ability to form phloem and xylem cell types. The vascular stem cells include spindle-shaped primitive cell and ray-shaped primitive cell (**Figure 1**) (Wybouw et al., 2024). Spindle-shaped primary cell produce secondary xylem inwards and secondary phloem outwards. Ray-shaped primitive cells produce wood rays inward and bast rays outward. These two kinds of cells constantly promote the radial growth of plants through peripheral division, that is to say, they form wood widely used in life. The vascular cambium is a crucial component of plant growth and development, particularly in the formation of secondary xylem and phloem tissues (Randall et al., 2015; Wang et al., 2021). It serves as a lateral meristem, producing secondary growth in stems and roots, contributing to radial thickening and the strengthening of plant axes.

The regulatory programs governing the development and functions of the vascular cambium have been a subject of extensive research, highlighting the role of various genes, hormones, and signaling pathways in its activity (Lee et al., 2021; Zheng et al., 2021; Sugimoto et al., 2022). For instance, studies have shown that cytokinins and auxins play interconnected roles in stimulating cambial activity, with an auxin concentration gradient across the cambium area crucial in regulating the process (Immanen et al., 2016; Zheng et al., 2021). Additionally, the TDIF-TDR-WOX4 signaling pathway has been identified as crucial in maintaining vascular meristem organization during secondary growth (Etchells et al., 2016). Furthermore, the genetic regulation of cambial development has been a focus, with genes such as *WOX4-like* genes and *MADS-box* genes identified as key regulators of cambial cell division activity and secondary growth in trees (Kucukoglu et al., 2017; Zhu et al., 2018). Moreover, epigenetic remodeling has been observed during vascular cambium periodicity, indicating the complexity and dynamic nature of vascular cambium activity. The interplay of various transcription factors and hormonal signals has been found to regulate vascular cambium activity, contributing to the production of vascular tissues essential for plant function (Lee et al., 2021). In conclusion, the vascular cambium plays a pivotal role in plant growth and development, particularly in the production of secondary xylem and phloem tissues. Its activity is regulated by a complex interplay of genetic, hormonal, and epigenetic factors, highlighting its significance in the overall development and functioning of plants.

## Regulation of hormone signaling pathway on division and differentiation of vascular cambium

Over the past decades, our understanding of the key molecular regulators that control vascular development has been steadily increasing with the application of biochemical, genetic, and genomic strategies (Ramachandran et al., 2020). In many cases, phytohormones, small peptides, and related transcription factors as indispensable regulatory factors in vascular development have been revealed. These hormones act in concert to tightly regulate the activities of the vascular cambium (Fischer et al., 2019). Auxin and cytokinin are intimately connected to virtually all developmental processes (**Supplementary Table 1**). *In vitro* experiments show that vascular cambium tissue can maintain the ability of autonomous division for a certain period of time, but the loss of primary meristem leads to the loss of its differentiation ability (Cho et al., 2017; Sun et al., 2023). Metabolic analyses showed that several phytohormones are preferentially enriched in distinct vascular tissue types. These results indicate that the activity of cambium and the differentiation of cambium stem cells are regulated by signals from the apical meristem, and phytohormones are the signal molecules between them.

### Auxin

More and more studies have confirmed that the regulation of vascular cambium activity is inseparable from the polar transport of auxin, and stem tips and leaves are the main parts of auxin synthesis (Du et al., 2020). It was found that when the shoot tip was removed, the plant would stop its secondary growth, but the exogenous auxin was applied to restore its secondary growth, which indicated that auxin was essential for the division and differentiation of vascular cambium cells (Agusti et al., 2011). In roots, the concentration of auxin reached the highest in cambium, followed by elongation zone, and the lowest in mature zone, indicating that auxin gradient is likely to provide location information for tissue differentiation. However, scientists have found that the maximum reactivity of auxin is not co-located with the maximum concentration of auxin (Smetana et al., 2019). In addition, the concentration of auxin is related to the fate of cells. High concentration of auxin can maintain the meristem of cells, and low concentration of auxin will lead to its differentiation into xylem or phloem (Bhalerao and Fischer, 2014). Baba et al (Baba et al., 2011)found that reducing the reactivity of auxin interferes with auxin signal pathway, which will lead to the decrease of vascular cambium division activity of poplar. Transcriptome analysis showed that during the short-term induction of plant growth stop, cambium dormancy was related to auxin response, not auxin content.

The importance of auxin to cambium development has been verified by functional research, and its mechanism in vascular cambium activities involves complex signal transduction and distribution patterns, which leads to the loss of radial stem growth, and emphasizes the key role of auxin in promoting cambium activities and subsequent wood formation (Motte et al., 2019). In addition, the regulation of auxin homeostasis regulates the activity and secondary growth of vascular cambium, which further emphasizes the complex control of auxin in cambium function (Lv et al., 2020). In addition, the dual regulation of auxin-mediated modules on xylem formation in poplar highlights the special role of auxin in coordinating wood formation through cambium activities (Tang et al., 2020).

In a word, the mechanism of auxin in vascular cambium activity involves complex signal and distribution characteristics. Many plant hormones and regulatory genes affect the reactivity and steady state of auxin to coordinate cambium function and wood formation.

### Gibberellins

As an important plant hormone, gibberellin participates in many biological processes such as plant growth and development, and cooperates with auxin in the secondary growth process of plants. Applying GAs to poplar can promote the division of cambium cells and enhance polar transport, thus increasing the level of auxin in stems. The regulation of auxin on xylem is caused by the promotion of GAs to a certain extent (Ragni et al., 2011). In the downstream of its reaction pathway, 83% of poplar genes affected by GAs are changed by auxin. In addition, by enhancing the response of auxin and the expression of auxin polar transporter PIN1 (Mäkilä et al., 2023), the establishment of cambium during poplar peeling and regeneration is promoted, indicating that there is a wide interaction between these two hormones (Björklund et al., 2007).

Under the background of vascular cambium activity, gibberellin has been proved to promote the branching of perennial woody plants (Ni et al., 2015), indicating that they participate in the regulation of woody growth process. In addition, exogenous gibberellin can change the morphology, anatomy and transcription regulation network of hormones in root and bud tissues, which further highlights the influence of gibberellin on plant growth and development. Prior to this, GA has been proved to increase the xylem formation of hypocotyl during flowering in Arabidopsis thaliana. In order to understand the role of heredity in the growth kinetics of cambium (Mäkilä et al., 2023), the researchers analyzed the roots in the early stage of secondary growth at cell resolution, which only produced two types of xylem cells: secondary xylem vessels and xylem parenchyma. GA treatment led to the increase of the number of secondary xylem vessels and xylem parenchyma, and the expansion of secondary xylem vessels increased, so GA promoted the production of xylem vessels and parenchyma in the early stage of root secondary growth. In a word, the mechanism of gibberellins in vascular cambium activities involves their regulation on stem elongation, branch branching, hormone interaction and transcription network controlling wood formation.

### Cytokinins

Cytokinins (CTKs) play a central role in the initiation and development of stratification, which can promote the fine cell division of vascular trunk. IPT is the rate-limiting enzyme in the synthesis of CTKs. In Arabidopsis tetraploid mutant ipt1/ipt3/ipt5/ipt7, the level of CTKs decreased, and cambium could not be formed, resulting in the decrease of the diameter of stems and roots (Yang et al., 2022). Applying exogenous CTKs to the mutant can restore its normal phenotype without affecting its vascular pattern. Therefore, CTKs can regulate the rate of cambium cell division without affecting the direction and form of cell differentiation. Werner have shown that cytokinin can stimulate vascular differentiation in the early stage, which plays a role in promoting cambium activity and subsequent wood formation (Werner et al., 2003). Furthermore, cytokinins have been implicated as central regulators of cambial activity, directly influencing the development and function of the vascular cambium (Bishopp et al., 2011). Additionally, transcriptomic analyses have provided insights into the regulatory role of cytokinins in cambial development, highlighting their significance as major hormonal regulators required for cambial activity.

Moreover, the distinct but interconnected distribution and signaling profiles of cytokinins and auxin have been reported to stimulate cambial activity, emphasizing the coordinated action of these hormones in regulating cambium function (Immanen et al., 2016). The role of cytokinin signaling in promoting cell division and activating the mitotic cell cycle further underscores its influence on cambial activity and secondary growth (Randall et al., 2015). Additionally, the long-distance basipetal transport of cytokinin has been demonstrated to control polar auxin transport and maintain the vascular pattern in the root meristem, indicating the systemic impact of cytokinins on vascular development (Bishopp et al., 2011).

Furthermore, the ectopic activation of cytokinin signaling at sites where protoxylem would normally differentiate highlights the specific role of cytokinins in coordinating hormonal interactions within the cambial microenvironment. The signaling transduction pathways of auxin and cytokinin leading to vascular cambium formation have been emphasized as essential for understanding vascular cambium development, further underscoring the significance of cytokinins in this process (Ye, 2002).

In conclusion, the mechanism of cytokinin in vascular cambium activity involves its regulatory effects on vascular differentiation, cell division, hormonal interactions, and the maintenance of vascular patterning, highlighting its multifaceted role in cambial development and wood formation.

### Strgolactone

In the process of secondary growth, the meristem cells in the cambium either maintain the stem cell population through proliferation or differentiate to form xylem or phloem. The balance between these two developmental trajectories is regulated by many environmental and internal factors. Strigolactone is a new plant hormone, which plays a key role in plant morphogenesis by inhibiting the growth of lateral buds, and at the same time regulates many growth and development processes such as plant height, light morphogenesis, leaf shape, anthocyanin accumulation, root morphology and plant adaptation to environmental stresses such as drought and low phosphorus (Wang et al., 2020a) Previous studies have shown that SLs can regulate the secondary growth of plants by promoting cambium activity. BES1 (Hu et al., 2022), a transcription factor downstream of SL signal transduction pathway, can promote shoot branching and xylem differentiation, and at the same time inhibit the expression of *WOX4*, a key factor regulating cambium division in TDIF-TDR signal transduction pathway. The antagonism between *BES1* and *WOX4* in the regulation of cambium activity may integrate the intercellular TDIF signal, thus efficiently regulating the proliferation and differentiation of cambium cells in both directions.

In addition, SLs signal will interact with auxin signal, which positively regulates cambium activity and is conservative among species (Bürger and Chory, 2020). Earlier studies reported that the regulatory effect of SLs on cambium may occur downstream of auxin signal (Hayward et al., 2009). The mutant of SLs biosynthesis genes (*MAX1, MAX3 and MAX4*) and the mutant of SLs signal molecule *MAX2* decreased the secondary growth in stems, but in Arabidopsis max mutant, the expression of *PIN1* and *PIN* increased, and the cambium activity decreased significantly. The application of *GR24*, an SLs analog, could induce the secondary growth, indicating that the effect of SLs on cambium activity may be caused by the change of auxin transport.

### Braassinosteroid

Brassinosteroid (BR) has the effect of promoting the division of the original cambium. In Arabidopsis, the number of vascular bundles was significantly increased in the stems of the mutants brassinosteroid insensitive 2 (*bin2*) and brassinazole-resistant 1-1D (*bzr1-1D*), while the number of vascular bundles was significantly decreased in the mutants of loss-of-function BR signaling bri1-116 and constitutive photomorphogenesis and dwarfism (*cpd*) (Ibanes et al., 2009). In addition to inducing cambium initiation at the primary growth stage, BR signaling also plays a role in regulating the type of vascular cambium differentiation. The differentiation ratio of phloem and xylem was distorted after mutation of the two BR receptors BRI-LIKE 1 (*BRL1*) and BRL3 in *Arabidopsis* (Lozano-Elena and Cano-Delgado, 2019). A similar phenomenon was observed in rice and poplar. Increased xylem differentiation in the gain-of-function mutant bri1-ethylmethylsulfone-suppressor1-D (*bes1-D*) of BR signaling (Kondo et al., 2014). A similar phenomenon has been observed in rice and poplar. After the inhibition of BR biosynthesis in poplar, the partial partialization of secondary lignosy decreased, and on the contrary, it increased when BR signal transduction was disturbed in rice (Du et al., 2020), the differentiation proportion of phloem in rice increased. In addition, there is a regulatory correlation between BR signaling and auxin signaling, and BR signaling and auxin coordinate the formation of vascular bundles (Nakamura et al., 2006; Lee et al., 2021). During vascular cambium cell division in tomato stems, BR signaling was closely related to local intracellular auxin homeostasis (Lee et al., 2021).

### Small peptide hormones

With the development of peptidomics, genomics, transcriptomics and genetics, there has been a great deal of research on the structure, classification and function of small peptide hormones in plants (Datta et al., 2024) They are characterized by low content, small molecular weight, large quantity, complex sources and processing mechanisms etc. Several families of small peptides that have received more attention in recent years are systemin (SYS) (Pearce et al., 1991), clavata 3/embryo surrounding region(CLE) (Goad et al., 2017), casprian strip integrity factor (CIF) (Nakayama et al., 2017), phytosulfokine (PSK) (Wang J. et al., 2015), and rapid alkalinization factor (RALFs) (Campbell and Turner, 2017; Wang J. et al., 2015). They act as an important signalling molecule that affects all stages of plant growth and development (**Table 1**). It allows both short-range cell-to-cell communication and long-range signaling. As a signaling molecule, small peptide hormones not only regulate physiological processes such as plant cell proliferation, tissue differentiation, organ formation, reproductive development, maturation and senescence, but also respond to plant responses to adversity stresses such as pests and diseases, droughts, cold, and salt stress. In conclusion, plant small peptide hormones are an emerging and highly promising area of research. It is believed that in the future, plant small peptide hormone can achieve quantitative production, so as to use fertilizer to regulate the growth of plants, improve the immune ability of plants to diseases and insect pests, and yield, solve the actual life and production problems, and provide us with great convenience.

**Table 1:** Function of small peptide hormones.

Family	Function	References
Systemin (SYS)	Responses that mediate plant defense	(Constabel et al., 1998; Bucherl et al., 2017)
Clavata 3/embryo surrounding region(CLE)	Maintain the homeostasis of the shoot apical and apical meristems Promote the proliferation of protocambial cells and inhibit their differentiation into xylem sieve tubes	(Fletcher et al., 1999; Rodriguez-Villalon, 2016; Goad et al., 2017; Fletcher, 2020; Reichardt et al., 2020)
Casprian strip integrity factor (CIF)	Regulating the integrity of the Kay belt Regulation of tapetum development and pollen wall formation	(Fujita, 2021; Truskina et al., 2022)
Phytosulfokine (PSK)	Regulating elongation of root and hypocotyl cells, pollen tubes, long, somatic embryogenesis, *in vitro* regenerative capacity, and legume nodulation processes; regulating the immune response of biotrophic and necrotic pathogens	(Yang et al., 2000; Wang C. et al., 2015; Stuhrwohldt et al., 2021)
Rapid alkalinization factor (RALFs)	Suphibit lateral root development; root growth and cell elongation Regulating lateral root development; causing pollen tube rupture to release sperm to complete double fertilization	(Matos et al., 2008; Gonneau et al., 2018; Ge et al., 2019; Blackburn et al., 2020)

### Other hormones

Auxin, gibberellin, cytokinin and strigolactone are all considered as growth hormones. In addition, ethylene and jasmonic brassinosteroid (BRs) can also regulate the activity of vascular cambium (Wang, 2020) (**Table 2**). Poplar was treated with ethylene and its precursor aminocyclopropane -1- carboxylate (ACC). It was found that both of them could promote cambium division and wood formation, and transgenic plants overexpressing ACC oxidase could also improve cambium activity and promote secondary growth. The number of vascular cells in the lower ring axis and inflorescence stem of *A.thaliana eto1* (Ethene over producer 1) mutant increased, while the number of vascular cells in the vascular tissue decreased during the secondary growth. Some studies found that there was also information exchange between ethylene signal pathway and TDIF-PXY signal (Etchells and Turner, 2010). JAs signaling pathway can also promote secondary growth. JAZ7 (Jasmonate-Zim-domain protein 7) and JAZ10 are negative regulatory factors in JAs signaling pathway, and their mutations can accelerate the initiation of cambium and make the stem thicker (Sehr et al., 2010). BRs is a key signal to promote the division of procambium during the primary growth of plants, but the molecular mechanism of its regulation is still unclear. Wang et al (Wang et al., 2022). used genome editing technology to study the homologous gene *PdBRI1s* of BRs receptor in poplar, and found that *PdBRI1s* controlled the initiation of stem cambium and xylem differentiation of poplar, but did not affect cambium division. The interaction between different plant hormones makes their role in regulating cambium activity very complicated, and more biochemical or molecular experiments are needed to reveal the mechanism of plant hormones in the future. All the above studies show that plant hormones can promote cambium activity, and whether there are some unknown negative regulatory signals in the regulation process still needs further study.

**Table 2:** Summary of plant hormones, their role in relation to tree-ring formation.

Plant hormone	Roles	References
Auxin (IAA)	Auxin gradient may provide location information for tissue differentiation, and the concentration of auxin is related to the fate of cells. High concentration of auxin can maintain cell meristem, while low concentration of auxin will lead to its differentiation into xylem or phloem	(Bhalerao and Fischer, 2014; Bürger and Chory, 2020)
Gibberellins (GAs)	Promote the division of cambium cells and enhance polar transport; Regulate xylem division and differentiation	(Israelsson et al., 2005; Jasinski et al., 2005; Tal et al., 2016; Ikematsu et al., 2017; Johnsson et al., 2019)
Cytokinins (CTKs)	Plays a central role in the initiation and development of cambium, which can promote the division of vascular stem cells	(Randall et al., 2015; De Rybel et al., 2013; Smet et al., 2019)
Strigolactones (SLs)	Positive regulation of cambium activity and conservation among species	(Hayward et al., 2009; Bürger and Chory, 2020)
Ethylene	Promote cambium division and wood formation	(Etchells and Turner, 2010; Etchells et al., 2012)
Jasmonic acid (JAs)	Promote secondary growth, accelerate the start of cambium, and thicken the stem.	(Sehr et al., 2010)
Brassinosteroid (BRs)	Critical signals to promote procambium division during plant primary growth	(Wang et al., 2022)

## Interaction between long-range signals (hormones) and short-range signals (peptides)

The interaction between hormones also affects cambium activity. There is an interactive feedback pathway between CTKs and auxin, which involves multi-layer transcription regulation and may help to determine the boundary between cambium and differentiated xylem. CTKs can guide auxin to flow to xylem by controlling the expression of *PIN1* and *PIN7*, and auxin can induce the expression of CTKs signal transduction inhibitor gene *AHP6* (Bishopp et al., 2011). The analysis of hormone distribution and genome-wide gene expression in the cambium showed that the content and signal level of CTKs in the cambium of transgenic poplar increased, as well as the concentration of auxin and the expression of response genes, which indicated that CTKs signal regulated cambium activity by affecting the concentration of auxin (Immanen et al., 2016).

GAs and auxin also have synergistic effect in the development of cambium. GAs stimulates the proliferation of cambium cells by promoting polar transport of auxin. In addition, the transcription group of poplar treated with GAs and auxin is highly heavy (Zhang et al., 2024), which indicates that there is extensive information exchange between these two hormones. In the development process of meristems in plants, auxin and cytokinin jointly regulate the maintenance and development of meristems through phase, mutual synergy or antagonism, and promote or inhibit SAM and RAM by regulating the different levels of auxin/cytokinin (Li et al., 2022). During the development of the whole meristem, ABA, GA, JA, SLs and so on respond to signaling pathways by cooperating or antagonizing the synthesis and transport of auxin and cytokinin, so as to realize the regulation of meristem development (Zheng et al., 2021).

**Figure 2:**
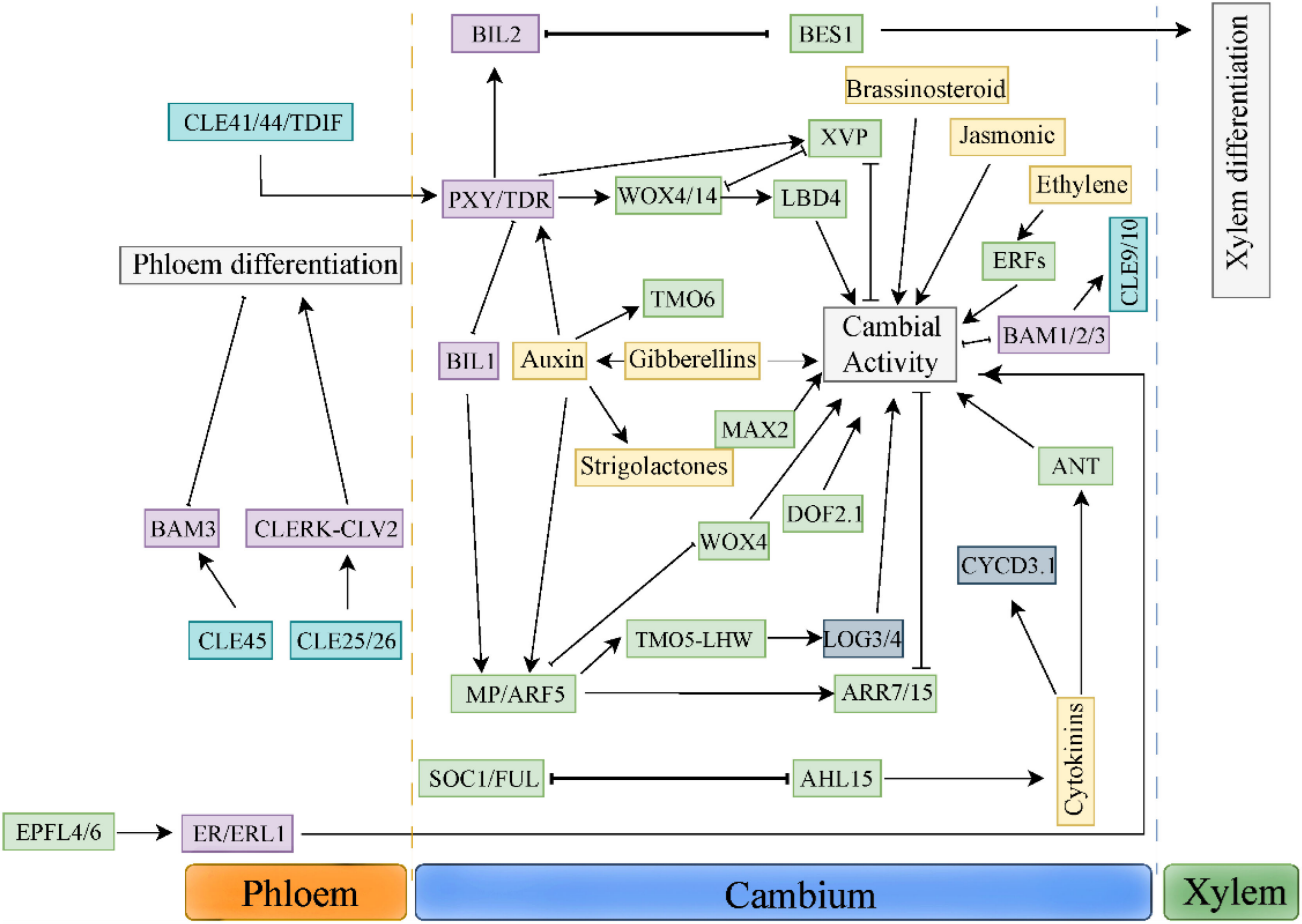
Synergistic actions among multilayered signals controlling cambium development.

There is a complex interaction between long-distance hormone signals and polypeptide signals (**Figure 2**). In this role, TDIF-PXY signal as a hub integrates various hormone signals, such as auxin, brassinolide and ethylene signal. *Bi2-like* (brassinosteroid-insensitive 2-like 2), a member of glycogen synthase kinase 3(GSK3) family, binds to PXY/TDR receptor, and prevents the differentiation of cambium cells into xylem by inhibiting transcription factor BES1(BRI1-EMS SUPPRESSOR 1) (Kondo et al., 2014). Further studies have found that both BES1 and VND6 transcription factors play a role in the downstream of GSK3s (Kondo et al., 2015) Studies have shown that MP/ARF5 can directly bind to the WOX4 promoter, reducing its expression in the cambium (Brackmann et al., 2018). BIL1 inhibits the activity of cambium by phosphorylating ARF5/MP, thus up-regulating the expression of *ARR7* (Arabidopsis response regulator 7) and *ARR15*, the negative regulators of CTKs signal. PXY/TDR can inhibit the activity of BIL1, thus weakening the effect of MP/ARF5 on the expression of *ARR7* and *ARR15* and increasing the activity of vascular cambium (Han et al., 2018). Ethylene can promote cambium activity and interact with TDIF-PXY signaling pathway (Yang et al., 2020). TDIF/PXY signaling pathway inhibits the expression of ethylene signaling pathway related genes ERFs, such as *BAM1*, *ERF018* and *ERF109*. In *pxy/tdr* and *wox4* mutants. The expression levels of *BAM1*, *ERF018* and *ERF109* were significantly up-regulated. In addition, compared with the hybrid plants of *erf109 erf018* and the single mutant of *pxy*, *pxy* showed more serious defects of cambial cell division, which indicated that ethylene and TDIF/PXY signaling pathway jointly regulated cambial cell division (Etchells et al., 2012). In a word, the interaction between *TDIF-PXY* and plant hormones is helpful for plants to better perceive and respond to long-distance development and environmental signals.

## Regeneration of the vascular cambium

The development of the vascular cambium begins with vascular stem cells that are capable of self-renewal and differentiation. In recent years, the molecular mechanisms of vascular cambium development have been greatly advanced through the study of model plants such as *Arabidopsis thaliana* (Zhang et al., 2019) and *Populus* (Chen et al., 2019). It has been found that the proliferation and differentiation of the vascular cambium is regulated by a variety of signals, including long-distance hormone signals (e.g., auxin, gibberellin, cytokinin, etc.), peptide signals, and transcription factors and microRNAs (miRNAs). These signaling pathways interact and coordinate with each other to regulate the activity of the vascular cambium.

Hormones such as auxin, gibberellins and cytokinins play important roles in vascular cambium regeneration. For example, auxin affects vascular cambium activity by regulating cell division and differentiation. Cytokinins, on the other hand, accumulate mainly in the secondary phloem and regulate vascular cambium activity in a non-cell-autonomous manner.

Recent studies have shown that epigenetic modifications, such as histone acetylation, are also involved in regulating vascular cambium development. The transcription factor PtrWOX4a and histone modification systems were found to synergistically regulate the development of the vascular cambium in *Poplar*. Specifically, *PtrVCS2* dynamically regulates the acetylation level of the *PtrWOX4a* gene by interacting with the histone acetylase complex, thereby controlling its expression and thus affecting vascular cambium development (Dai et al., 2023). Zhou et al. resolved the mechanism of action of histone deacetylase (HDAC) in specifically removing acetylation modifications of cytoplasmic metabolic enzymes and transcription factors (Xu Q, et al., 2021; Xu C, et al., 2021) (**Supplementary Figure 1**). In addition, miRNAs such as *miR156*、*miR172*、*miR396*、*miR529* and *miR164* are also involved in regulating the regeneration process of the vascular cambium (Song et al., 2014; Qiu et al., 2015). These modifications affect the activity of the vascular cambium by influencing gene expression and, consequently, the activity of the vascular cambium (Inacio et al., 2022).

Despite significant progress in vascular cambium regeneration, numerous questions remain to be addressed. For instance, the processes controlling vascular stem cell maintenance and proliferation of the vascular cambium are still poorly understood. Furthermore, many of the factors influencing secondary growth exhibit tissue- and time-specificity, necessitating careful consideration of these properties in future investigations of gene function. To address these challenges, the integration of depth imaging techniques (Wang et al., 2020b) with gene expression analysis offers a promising approach to characterize the initiation and development of formation layer cells. This combination provides a novel tool for advancing our study of vascular cambium regeneration, enabling a deeper understanding of the underlying mechanisms.

## Conclusion and prospect

The activity of vascular cambium in plants produces vascular tissue, which runs through the whole plant and participates in the transportation, support and consolidation of substances in plants. Vascular cambium has always been a problem that researchers pay close attention to. It is of great significance to explore the development process of plant vascular cambium, which helps to explain the molecular mechanism of plant genetic evolution, growth and development and adaptation to the environment. In recent years, the molecular regulation of plant vascular cambium has made a breakthrough, from a single regulatory pathway initially discovered to the current network pathway of environmental, hormonal and molecular regulation. By summarizing the research progress of plant hormones regulating the division and differentiation of vascular cambium, we can deeply understand the mechanism of hormone signal regulation network in vascular cambium, and provide scientific basis for agricultural production and plant breeding. However, compared with apical meristem, people know little about the control process of vascular stem cell maintenance and cambium proliferation. In the future, we should strengthen the in-depth research on the interaction and regulation of various network pathways. In addition, because many factors affecting secondary growth are tissue-specific feature research must consider tissue-specific and time-specific to study gene function.

Conservation and specificity can be revealed by comparing the similarities and differences in the regulatory mechanisms of the vascular formation layer in different species. It has been shown that there are conserved regulatory mechanisms for vascular formation layer activities among species, and these include interactions of hormonal signaling (e.g., auxin, gibberellin, etc.), peptide signaling, transcription factors, and microRNAs (Shimadzu et al., 2023). However, there are significant differences in the structure and cellular events of the vascular formation layer in different plants, e.g., the arrangement of the vascular formation layer primordia and the cellular morphology differ in monocotyledonous and dicotyledonous plants (Jura-Morawiec et al., 2021). In addition, some plants such as Calycopteris floribunda and Dalechampia coriacea exhibit distinctive patterns of secondary vascular bundles development, suggesting the existence of specific regulatory mechanisms in some species (Rajput et al., 2022). Overall, despite the existence of conserved regulatory networks, different species show significant specificity in the regulatory mechanisms of vascular formation layers.

However, because of the long growth cycle, high genetic heterozygosity and difficult genetic analysis, it is difficult to change its characters to meet different test requirements by using the existing technology. To sum up, only by finding the key genes of vascular cambium development can we achieve accurate regulation at the molecular level, thus improving forest characteristics. Deep imaging combined with gene expression analysis technology is helpful to characterize the start-up and development of cambium cell (Oralová et al., 2019), and mathematical modeling and simulation combined with real-time imaging have important application prospects in simulating cambium growth and potential molecular processes (Bencivenga et al., 2016). With depth imaging, the initiation and development of cells in the formation layer can be characterized, while mathematical modeling and simulation are able to construct a regulatory framework for formation layer growth, simulate the localization and distribution of formation layer tissues in trees, and reveal the communication between tissues and cells (Yankeelov et al., 2010; Sozzani et al., 2014) (Yankeelov et al., 2010; Sozzani et al., 2014). The combination of these techniques will not only help to comprehensively analyze the molecular mechanism of vascular formation layer development in woody plants, but also provide theoretical support for future molecular breeding of forest trees (Dapšys and Čiegis, 2023). At present, the tissue section and imaging analysis required by this technique have been applied to *Arabidopsis* hypocotyl, and this technique should be applied to forest research in the future. With the development of genomics and functional genomics, it is of great significance to screen candidate genes in the whole genome by using the existing research results of vascular cambium transcriptome to reveal the complex mechanism of plant hormones regulating vascular cambium. Further revealing the intricate hormone regulation network in vascular cambium and realizing the artificial regulation of the secondary growth and development will undoubtedly pave the way for innovative strategies in agricultural production and plant breeding, and further realize the ultimate goal of wood properties improvement.

## Supplementary material

Supplementary Table 1 The roles of various growth hormones during the development of the vascular cambium.

Supplementary Table 2 The full names of gene(s) list which are mentioned in the manuscript.

Supplementary Figure 1 Histone deacetylase regulates ribosomal proteins.
