# Supplementary material for: Phytohormones involved in vascular cambium activity in woods: current progress and future challenges
Source: Front Plant Sci. 2024 Dec 17;15:1508242. doi: 10.3389/fpls.2024.1508242 (PMC11685017; doi:10.3389/fpls.2024.1508242)
Supplement: Supplementary Table 1 — The roles of various growth hormones during the development of the vascular cambium. [file Table1.docx]

| **Developmental Process** | **Role of Auxin** | **Role of Cytokinin** | **References** |
| --- | --- | --- | --- |
| Apical Bud Dominance | Promotes cell elongation in the shoot apex, inhibiting bud outgrowth below the apex | Stimulates lateral bud growth when levels are high relative to auxin | (Turchi et al., 2015; Zhang et al., 2020) |
| Root Initiation and Development | Induces root formation and controls root architecture | Promotes root hair development and lateral root initiation | (Wu et al., 2021a) |
| Shoot Elongation | Stimulates cell elongation in the stem | Inhibits shoot elongation when in high concentration relative to auxin | (Hurny et al., 2020) |
| Leaf Formation | Regulates leaf primordia initiation and growth | Influences leaf morphogenesis and senescence | (Wu et al., 2021b) |
| Flowering | Triggers the transition to flowering in some plants | Can delay flowering, involved in the regulation of flowering time | (Zhao et al., 2010) |
| Fruit Development | Influences fruit set and growth | Involved in fruit development and ripening processes | (Grosskinsky and Petrasek, 2019) |
| Vascular Tissue Differentiation | Promotes xylem differentiation | Promotes phloem differentiation and maintenance | (Rong et al., 2022) |
| Response to Gravity (Gravitropism) | Controls the direction of root growth in response to gravity | Involved in the gravitropic response, balances auxin action | (Nakamura et al., 2019) |
| Response to Light (Phototropism) | Mediates phototropic bending of stems towards light source | Influences phototropic responses, sometimes counteracting auxin | (Fankhauser and Chory, 1997; Zhu et al., 2008) |
| Wound Healing and Tissue Repair | Promotes cell division and regeneration at wound sites | Stimulates cell division and proliferation in healing tissues | (Pawełkowicz et al., 2024) |
| Senescence and Plant Aging | Regulates the aging process and senescence of plant organs | Can delay senescence when in higher concentration | (Glanz-Idan et al., 2022) |

**Supplement Table 1**．The roles of various growth hormones during the development of the vascular cambium.

**References:**

Fankhauser, C., and Chory, J. (1997). Light control of plant development. *Annu. Rev. Cell Dev.Biol.* 13, 203-229. doi:10.1146/annurev.cellbio.13.1.203

Glanz-Idan, N., Lach, M., Tarkowski, P., Vrobel, O., and Wolf, S. (2022). Delayed leaf senescence by upregulation of cytokinin biosynthesis specifically in tomato roots. *Front. Plant Sci.* 13, 922106. doi:10.3389/fpls.2022.922106

Grosskinsky, D.K., and Petrasek, J. (2019). Auxins and cytokinins - the dynamic duo of growth-regulating phytohormones heading for new shores. *New Phytol.* 221, 1187-1190. doi:10.1111/nph.15556

Hurny, A., Cuesta, C., Cavallari, N., Otvos, K., and Duclercq, J., et al. (2020). SYNERGISTIC ON AUXIN and CYTOKININ 1 positively regulates growth and attenuates soil pathogen resistance. *Nat. Commun.* 11, 2170. doi:10.1038/s41467-020-15895-5

Nakamura, M., Nishimura, T., and Morita, M.T. (2019). Gravity sensing and signal conversion in plant gravitropism. *J. Exp. Bot.* 70, 3495-3506. doi:10.1093/jxb/erz158

Pawełkowicz, M., Zieniuk, B., Staszek, P., and Przybysz, A. (2024). From sequencing to genome editing in cucurbitaceae: application of modern genomic techniques to enhance plant traits. *Agriculture* 14, 90. doi:10.3390/agriculture14010090

Rong, C., Liu, Y., Chang, Z., Liu, Z., and Ding, Y., et al. (2022). Cytokinin oxidase/dehydrogenase family genes exhibit functional divergence and overlap in rice growth and development, especially in control of tillering. *J. Exp. Bot.* 73, 3552-3568. doi:10.1093/jxb/erac088

Turchi, L., Baima, S., Morelli, G., and Ruberti, I. (2015). Interplay of HD-zip II and III transcription factors in auxin-regulated plant development. *J. Exp. Bot.* 66, 5043-5053. doi:10.1093/jxb/erv174

Wu, L., Wang, J., Li, X., and Guo, G. (2021a). Cytokinin-controlled gradient distribution of auxin in arabidopsis root tip. *Int. J. Mol. Sci.* 22. doi:10.3390/ijms22083874

Wu, L., Wang, J., Li, X., and Guo, G. (2021b). Cytokinin-controlled gradient distribution of auxin in arabidopsis root tip. *Int. J. Mol. Sci.* 22. doi:10.3390/ijms22083874

Zhang, Y., Rodriguez, L., Li, L., Zhang, X., and Friml, J. (2020). Functional innovations of PIN auxin transporters mark crucial evolutionary transitions during rise of flowering plants. *Sci. Adv.* 6. doi:10.1126/sciadv.abc8895

Zhao, Z., Andersen, S.U., Ljung, K., Dolezal, K., and Miotk, A., et al. (2010). Hormonal control of the shoot stem-cell niche. *Nature* 465, 1089-1092. doi:10.1038/nature09126

Zhu, D., Maier, A., Lee, J., Laubinger, S., and Saijo, Y., et al. (2008). Biochemical characterization of arabidopsis complexes containing CONSTITUTIVELY PHOTOMORPHOGENIC1 and SUPPRESSOR of PHYA proteins in light control of plant development. *Plant. Cell.* 20, 2307-2323. doi:10.1105/tpc.107.056580
