# Supplementary material for: Phytohormones involved in vascular cambium activity in woods: current progress and future challenges
Source: Front Plant Sci. 2024 Dec 17;15:1508242. doi: 10.3389/fpls.2024.1508242 (PMC11685017; doi:10.3389/fpls.2024.1508242)
Supplement: Supplementary Table 2 — The full names of gene(s) list which are mentioned in the manuscript. [file Table2.docx]

**Supplement Table 2**．The full names of gene(s) list which are mentioned in the manuscript.

| **Gene name** | **Full names of gene** |
| --- | --- |
| *IPT* | Isopentenyl transferase |
| *PIN1* | PIN-FORMED 1 |
| *PIN7* | PIN-FORMED 7 |
| *AHP6* | *Arabidopsis* histidine kinase 6 |
| *BES1* | BRI1-EMS SUPPRESSOR 1 |
| *WOX4* | WUSCHEL-related homeobox 4 |
| *TDIF* | TRACHEARY ELEMENT DIFFERENTIATION INHIBITOR |
| *PXY* | PHABULOSA |
| *TDR* | PXY-related 1 |
| *ARF5/MP* | AUXIN RESPONSE FACTOR 5/Monopteros |
| *ARR7* | *Arabidopsis* response regulator 7 |
| *ARR15* | *Arabidopsis* response regulator 15 |
| *ERF018* | ETHYLENE RESPONSE FACTOR 018 |
| *ERF109* | ETHYLENE RESPONSE FACTOR 109 |
| *BAM1* | BRI1-ASSOCIATED MERISTEM 1 |
| *PtrVCS2* | *Populus trichocarpa* Vascular Cambium Stem Cell 2 |
| *PtrWOX4a* | *Populus trichocarpa* WUSCHEL-related homeobox 4a |
| *miR476a* | microRNA 476a |
| *Pd BRI1s* | *Populus* deltoides BRI1-like |
| *PdBRI1s* | *Populus* deltoides BRI1-like |
| *BIL1* | BI2-like 2 |
| *Dof2.1* | DNA-binding with one finger 2.1 |
| *Tmo5/Lhw* | TMO5/LHW |
| *SYS* | Systemin |
| *CLE* | CLAVATA 3/embryo surrounding region |
| *CIF* | Casprian strip integrity factor |
| *PSK* | Phytosulfokine |
| *RALFs* | Rapid alkalinization factor |
| *JAZ7* | Jasmonate-ZIM-domain protein 7 |
| *JAZ10* | Jasmonate-ZIM-domain protein 10 |
| *NPf3* | NPF3 |
| *PtrHB4* | *Populus trichocarpa* Homeobox gene 4 |
| *Ahd-zip iii* | Asymmetric leaf 1 |
| *NTL9* | NAC-like transcription factor 9 |
| *ACS7* | 1-aminocyclopropane-1-carboxylate synthase 7 |
