# Supplementary figures and images for: Phytohormones involved in vascular cambium activity in woods: current progress and future challenges
Source: Front Plant Sci. 2024 Dec 17;15:1508242. doi: 10.3389/fpls.2024.1508242 (PMC11685017; doi:10.3389/fpls.2024.1508242)

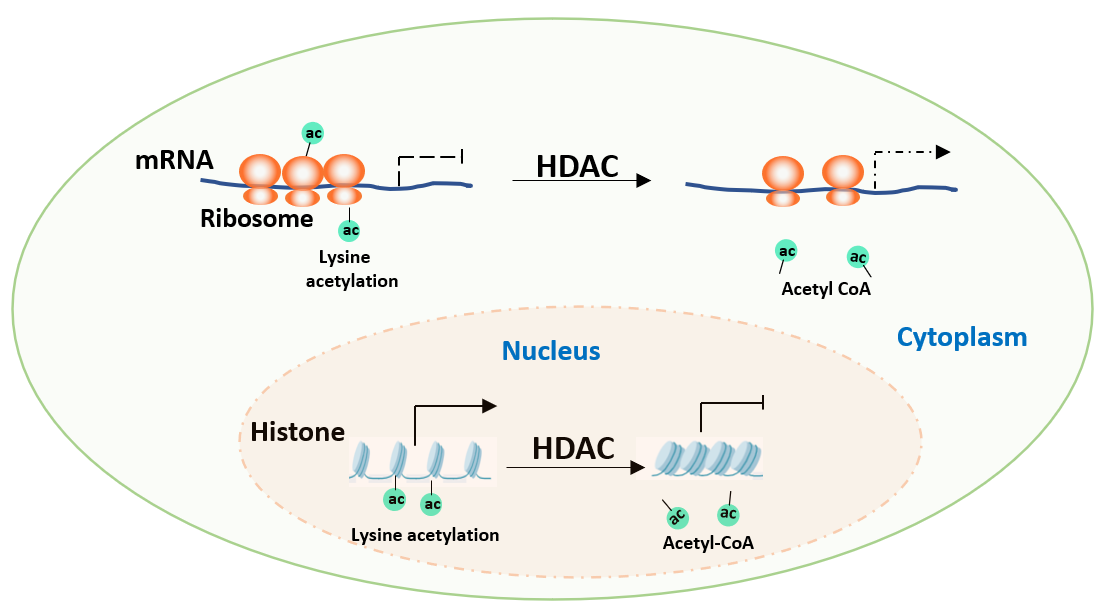

Supplement: Supplementary Figure 1 — Histone deacetylase regulates ribosomal proteins. [file Image1.png]
